# Outflow Tract Formation—Embryonic Origins of Conotruncal Congenital Heart Disease

**DOI:** 10.3390/jcdd8040042

**Published:** 2021-04-09

**Authors:** Sonia Stefanovic, Heather C. Etchevers, Stéphane Zaffran

**Affiliations:** Aix-Marseille Université, INSERM, MMG, U1251, 27 boulevard Jean Moulin, 13005 Marseille, France; heather.etchevers@inserm.fr

**Keywords:** congenital heart defects, cardiac progenitor cells, outflow tract, second heart field, neural crest, endocardium, cushion, valve

## Abstract

Anomalies in the cardiac outflow tract (OFT) are among the most frequent congenital heart defects (CHDs). During embryogenesis, the cardiac OFT is a dynamic structure at the arterial pole of the heart. Heart tube elongation occurs by addition of cells from pharyngeal, splanchnic mesoderm to both ends. These progenitor cells, termed the second heart field (SHF), were first identified twenty years ago as essential to the growth of the forming heart tube and major contributors to the OFT. Perturbation of SHF development results in common forms of CHDs, including anomalies of the great arteries. OFT development also depends on paracrine interactions between multiple cell types, including myocardial, endocardial and neural crest lineages. In this publication, dedicated to Professor Andriana Gittenberger-De Groot and her contributions to the field of cardiac development and CHDs, we review some of her pioneering studies of OFT development with particular interest in the diverse origins of the many cell types that contribute to the OFT. We also discuss the clinical implications of selected key findings for our understanding of the etiology of CHDs and particularly OFT malformations.

## 1. Introduction

Congenital heart defects (CHDs) are the most common form of birth defect, affecting up to 1 in every 100 live births. Anomalies in cardiac outflow tract (OFT) are among the most frequent CHDs, with a prevalence of 30%, likely reflecting the complex morphogenetic events underlying heart development [1,2]. The cardiac OFT, comprised of the *conus* or *bulbus cordis* and the *truncus arteriosus* (together called the conotruncal region), is a rapidly remodeling structure during embryogenesis at the arterial pole of the heart, as it connects the embryonic ventricles to the aortic sac [3]. The OFT forms during heart looping from progenitor cells in splanchnic pharyngeal mesoderm. At its maximal extension, the OFT is a torsioned myocardial cylinder lined with endocardial cells. Septation of the OFT, to give rise to the base (trunk) of the aorta and pulmonary arteries, involves interactions between multiple cell types, including myocardial, endocardial and neural crest cells. During this process, the myocardial wall of the OFT rotates prior to and during the septation of this region [4]. Two opposing endocardial cushions swell into the extracellular matrix (ECM) composing the cardiac jelly between the myocardial sleeve and the endocardial lining. These will form the major septal OFT cushions, designated as inferior or superior, at the origin of the semilunar (aortic and pulmonary) valves and the transition from heart to great arteries. OFT remodeling is associated with formation of an outlet septum, semilunar valve morphogenesis, coronary arteriogenesis and maturation of the myocardial and smooth muscle components at the base of the great arteries.

The septation of the OFT is established first by the fusion of the superior and inferior cushions at their distal end and proceeds proximally in the direction of the ventricles. Absence of OFT septation leads to persistent truncus arteriosus (PTA), while an unbalanced septation of the OFT can induce double outlet right ventricle (DORV), overriding aorta (OA), ventricular septal defect (VSD), or other anomalies such as tetralogy of Fallot (ToF). In addition, anomalies may be associated with abnormal rotation of the OFT, leading to a lack of alignment of the aorta or pulmonary artery with their respective ventricles [4]. These CHDs are frequently diagnosed in newborns with cyanosis. Here, we will discuss the embryological mechanisms of CHDs associated with OFT defects.

## 2. Embryonic Origins of the OFT

### 2.1. Myocardial Progenitors

Distinct sets of cardiac progenitors differentiate in a coordinated manner to form the different parts of the heart. Embryonic heart development starts from the earliest stages of gastrulation, around embryonic day (E) 6.5 in the mouse or within a few hours’ incubation of the chicken egg, with the specification of an initial pool of cardiac progenitors expressing the transcription factor Mesp1 [5,6]. These cells ingress and migrate from the primitive streak to the anterior part of the embryo, where within a few (chick) to 24 h (mouse), they form a “first heart field” (FHF) known as the cardiac crescent in mouse or a bilateral pair of primary heart fields in chick [7]. By E7.5–8.0, during folding of the mouse embryo and formation of the foregut, the two sides of the cardiac crescent are brought together to form the linear heart tube.

**Figure 1:**
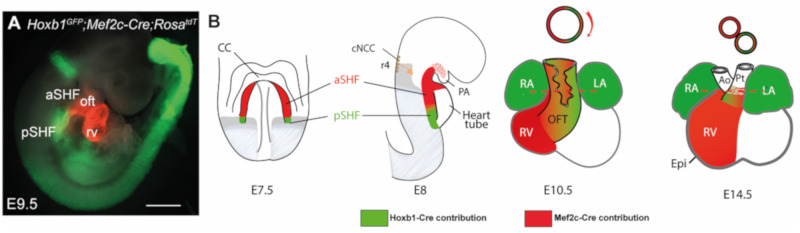
Subdomains within the SHF. (**A**) Whole-mount fluorescence microscopy of triple transgenic *Mef2c-Cre*;*Hoxb1-GFP*;*Rosa*^tdT^ embryos at stage E9.5. (**B**) Diagram showing stages of heart development and contribution of the anterior second heart field (red) and the posterior second heart field (green) to the heart. Frontal view is shown for embryonic day 7.5 (E7.5), E10.5, E14.5 and lateral view for E8. Genetic lineage analysis of *Hoxb1*-expressing cells using the *Hoxb1*-IRES-*Cre* mouse line showed that *Hoxb1*+ progenitors contribute to both atria, the DMP and the myocardium at the base of the pulmonary trunk. Genetic lineage analysis of *Mef2c-Cre*-labelled cells using the *Mef2c-Cre*;*Rosa*^tdT^ mouse line showed that Tomato-positive (Tomato+) cells are detected in the DMP, the great arteries (aorta and pulmonary trunk) and the right ventricle. The left ventricle is derived exclusively from the first heart field, which also contributes to atria formation. SHF, second heart field; Ao, aorta; CC, cardiac crescent; cNCC, cardiac neural crest cells; Epi, epicardium; LA, left atrium; LV, left ventricle; PA, pharyngeal arch; Pt, pulmonary trunk; RA, right atrium; RV, right ventricle; a/pSHF, anterior/posterior second heart field. Scale bar: 500 μm.

The embryonic myocardium of this initial tube is characterized by lower proliferation, a poorly developed contractile apparatus and slow conduction relative to later stages [8]. Rapid growth of the heart is driven by progressive addition of progenitor cells from adjacent pharyngeal mesoderm to its arterial and venous poles. These cells, collectively known as the second heart field (SHF), were first identified in the mouse and chick models [9,10] but appear to be present in all vertebrate embryos examined to date. SHF progenitors, located in a dorsomedial position relative to the linear heart tube, are kept in an undifferentiated and rapidly proliferating state. The SHF gives rise to right ventricular and OFT myocardium at the arterial pole, and to atrial myocardium including the dorsal mesenchymal protrusion (DMP) at the venous pole [10]. Perturbation of the SHF during elongation of the forming heart results in a spectrum of OFT defects associated with early embryonic lethality [11,12,13]. Lineage tracing analysis in mice has revealed that the SHF is sub-divided into distinct anterior and posterior regions (aSHF and pSHF) [14,15] (Figure 1). Indeed, aSHF progenitors engender the right ventricular and proximal OFT myocardium, while progenitor cells located in the pSHF contribute to the formation of the atrial and atrioventricular septation through development of the dorsal mesenchymal protrusion (DMP) that forms the muscular base of the primary atrial septum [16]. In addition, a subset of SHF cells form the myocardium at the base of the pulmonary artery [14]. Specific regions in the embryonic heart tube acquire a chamber-specific gene program, and from E8.5 on, differentiate and expand or “balloon” by rapid proliferation to form the ventricular and atrial chamber myocardium. In contrast, the regions between these differentiating chambers, such as the *sinus venosus*, the atrioventricular canal (AVC) and the OFT, either do not differentiate or expand during this temporal window, and consequently form constrictions. The inflow tract cells of the heart tube then develop into atrial cells, pulmonary myocardial cells and myocardial cells of the proximal superior caval veins.

The relevance of the SHF to congenital malformations has stimulated research into the mechanisms regulating the process of cardiac tube elongation. A complex network of signaling inputs and transcriptional regulators controls the elevated proliferation and differentiation delay relative to FHF cells. Identification of SHF-restricted regulatory elements has provided evidence that different transcriptional programs operate in distinct SHF sub-populations, giving rise to specific subregions of the definitive heart. Cells expressing *Cre* recombinase under the control of a SHF-restricted regulatory element from the *Mef2c* transcription factor gene contribute widely to the OFT and right ventricle, as well as to a population of cells at the venous pole of the heart giving rise to the primary atrial septum and the DMP [17,18,19,20]. Expression of the LIM homeodomain transcription factor Islet-1 (*Isl1*) in SHF cells showed that these progenitors contribute to the venous, as well as the arterial pole of the heart [13]. The secreted fibroblast growth factor 10, encoded by *Fgf10*, is also a marker of the murine aSHF and subsequent arterial pole myocardium [21]. The cardiac *ISL1* expression domain is conserved during human embryonic development; in early heart tissue, ISL1 binds a *FGF10* enhancer element [22]. Retinoic acid (RA) has been shown to pattern the SHF [23,24]. Genetic lineage analysis in the mouse has shown that combinatorial expression of anterior Homeobox (Hox) genes *Hoxa1, Hoxa3* and *Hoxb1* defines distinct sub-domains within the posterior domain of the SHF that contribute to most of the atrial and sub-pulmonary myocardium [14,25]. Thus, animal models are relevant to studying conserved molecular circuitry in regions prone to human CHDs.

Recently, we have used chromatin accessibility analysis with ATAC-seq (Assay of Transposase Accessible Chromatin sequencing) to define accessible sites for transcriptional regulation in SHF subpopulations [26]. The ATAC-seq method utilizes a transposase, which preferentially integrates sequencing adapters in regions of open chromatin such as active promoter regions, enhancers, and insulators, and can be performed on relatively small amounts of cells. Such datasets are important to understand the tightly regulated genetic networks governing heart development and to determine how these networks become deregulated in CHD. Integration with transcriptomes of sorted cardiac progenitor cells led to the discovery of novel pSHF markers (*Aldh1a2*, *Bmp4*, *Gata4*, *Nr2f2*, etc.) that contribute to the formation of the OFT and venous pole of the heart, illustrating how these datasets provide multiple new avenues of investigation for the future [26].

### 2.2. Endocardial Cushions

The heart tube is formed by both future ventricular and atrial myocardium and a non-chamber myocardial phenotype in the AVC, OFT and inner curvature [27]. This myocardium signals to underlying AVC and OFT endocardium to activate an endocardial-to-mesenchymal transition (EndMT), in which a subset of endocardial cells migrates into the underlying ECM to give rise to the mesenchyme of the valves and the septa [28]. Reciprocal signals between the endocardium and the myocardium have been shown to induce EndMT, mediated by the transforming growth factor β, bone morphogenetic protein (BMP) and Notch signaling pathways, among others [29,30]. Primary myocardium of the AVC and OFT is normally characterized by *Bmp2* and *Bmp4* expression [31,32], while *Bmp2*-deficient hearts lack AVC and OFT cushions [32,33,34]. Studies in mice have revealed that the AVC T-box transcription factors, Tbx2 and Tbx3, repress chamber-specific gene expression [32,33] and that Bmp2 directly regulates *Tbx2* expression in the cushion regions [35]. Notch signaling is crucial to patterning the forming heart and presumptive chamber and valve tissues by restricting *Bmp2* and *Tbx2* expression to the cushions [29]. Notch is activated throughout the endocardium of the AVC and OFT regions. Ectopic activation of *Notch2*, or the Notch target genes *Hey1* and *Hey2* in the chicken heart, results in downregulation of Bmp2 in the AVC [36], suggesting that the Notch pathway suppresses *Bmp2* expression in chamber myocardium. However, Bmp and Notch signals cooperate to induce EndMT in the AVC and OFT regions in mouse embryos [37]. Alterations of AVC and OFT cushion architecture result from ligation-induced changes in blood flow in the chicken embryo model, highlighting additional interactions with shear stress-responsive signaling pathways during the EndMT process [30].

**Figure 2:**
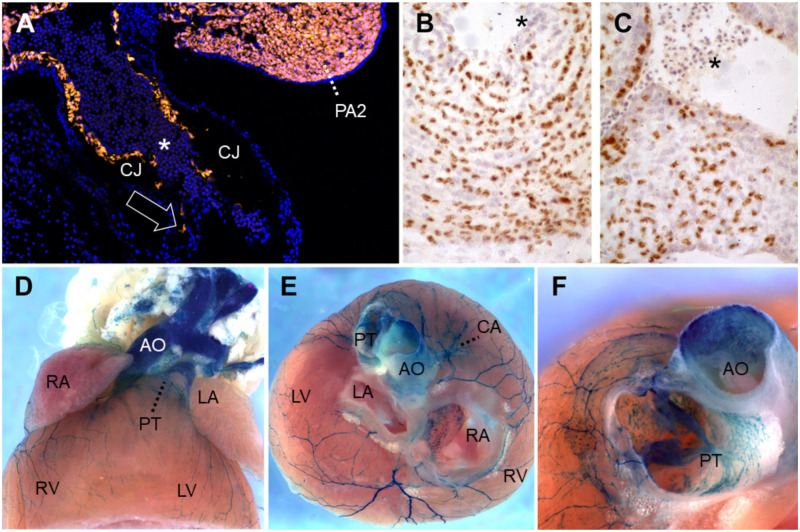
Lineage-tracing of neural crest cells in the cardiac outflow tract. (**A**) *Wnt1-Cre*-mediated recombination induces Tomato red fluorescent protein expression in neural crest-derived cells (NCC) of PA2 as well as in those (arrow) migrating caudally into the outflow tract, along the endothelial abluminal surface on embryonic day (E)10 in mouse. Blue, DAPI-stained nuclei. (**B**,**C**) Quail-chick chimeras with a unilateral isotopic and isochronic substitution of a rhombomere 8 neural fold at the level of somites 1–3 at the 5-somite stage. By embryonic day 8.5 (Hamburger-Hamilton stage 33), these demonstrate permanent immunoreactivity for the QCPN antibody, brown, in many of the NCC within the (**B**) proximal pulmonary trunk and (**C**) aortic valve leaflets. Hematoxylin counterstain. (**D**–**F**) *Wnt1-Cre*-mediated recombination induces b-galactosidase activity in the presence of Xgal substrate in NCC of the adult murine heart on postnatal day 50. (**D**) Ventral view. (**E**) After removing the atria, one observes labeled great artery smooth muscle as well as at the base of the coronary arteries, the large branches of autonomic nerves and numerous melanocytes lining the right interatrial septum around the *foramen ovale*, all of NCC origin. Melanocytes are also frequently seen along valve leaflets, *chordae tendinae*, on and within the interventricular septum and within the proximal great arteries [132,133,134,135,136]. Anterior view, ventral to top. (**F**) A slightly more ventral view of the same heart shows labeled cells within the *tunica media* of the murine ascending aorta and pulmonary trunk base, as well as in leaflets and mesenchyme of the pulmonary valve. All photos courtesy of the authors. (**B**) and (**C**) have been previously published as Figure 5K,L in Reference [52]. Asterisks: nucleated primitive erythroblasts. Abbreviations: AO, ascending aorta; CA, coronary artery; CJ, cardiac jelly; LA, left atrium; LV, left ventricle; PA2, pharyngeal arch 2; PT, pulmonary (arterial) trunk; RA, right atrium; RV, right ventricle; Xgal, 5-bromo-4-chloro-3-indolyl-β-D-galactopyranoside.

EndMT in the AVC and OFT regions is essential for generating a pool of mesenchymal cells that subsequently become valvular interstitial cells during cardiac valve maturation. However, other cell lineages contribute to endocardial cushions. In the AVC, lineage analysis using *Tie2-Cre;Rosa26R* mice has shown that the majority of mesenchymal cells are derived from endocardial cells following EndMT [38]. In contrast, lineage tracing studies of the OFT using quail-chick chimeras and, later, *Wnt1-Cre*-expressing mice, have demonstrated that cardiac neural crest cells (CNCC) contribute significantly to both arterial valves and the aorticopulmonary septum [38,39,40] (Figure 2). These respond directly to Bmp signaling. *Cre*-mediated *Bmpr1a* deletion in the CNCC lineage alone leads to hypoplasia of the early OFT cushions and retrograde, often lethal, diastolic arterial flow at E10.5–11.5. By E12.5, surviving mouse embryos also develop a persistent truncus arteriosus (PTA) [41]. Two recent studies have reported that the intercalated leaflets of the semilunar valves arise directly from aSHF progenitors of the outflow wall rather than indirectly after EndMT or from CNCC [42,43]. Thus, the mesenchymal cell populations forming the cushions come from multiple sources and coordinately direct blood outflow from the heart into the aorta and pulmonary trunk.

Recently, single-cell transcriptomic analysis generated a reference of cell transcriptomic states in OFT development [44]. This study identified convergent development of the vascular smooth muscle cell (vSMC) lineage, where intermediate cell populations were found to be involved in either myocardial-to-vSMC trans-differentiation or a mesenchymal-to-vSMC transition. The OFT myocardium derives from the SHF [45], so the vSMC derived from myocardial trans-differentiation are actually a small portion of SHF-derived vSMC, whereas most are derived from either SHF or CNCC-mesenchyme (see below). Transitions between cell lineages during OFT development are thus more complicated than previously appreciated.

### 2.3. Cardiac Neural Crest

The CNCC is a subpopulation of neural crest cells that contributes most cells of the perivascular walls of the posterior pharyngeal (aortic) arteries [46,47] and is also indispensable for septation of the more proximal OFT, as described briefly above. CNCC originate from dorsal neural folds corresponding to the prospective posterior hindbrain and first vertebral segments of spinal cord, at the boundary with non-neural ectoderm and at the head–neck interface. A broader term that includes the CNCC is the “vagal” neural crest, since these also provide glial and perineural support cells accompanying the vagal nerve to all its targets, many neurons of the corresponding cranial ganglia, and the autonomic enteric nervous system innervating the fore- and mid-gut (reviewed in Reference [48]).

CNCC migrate circumpharyngeally around the endoderm [49] in a dorsal-to-ventral manner, reflecting their temporal order of arrival rather than inherent differences in competence [50]. They also mix to a lesser extent along the rostro-caudal axis to adjacent pharyngeal arches [51,52] and do the same at the level of adjacent rostral somitic segments corresponding to the first vertebrae [53]. These dynamic scenarios were deduced from fate maps of painstaking quail-chick chimeras using microsurgery and experimental embryology, stemming from the first demonstrations fifty years ago that neural crest gives rise to mesenchyme (“mesectoderm”) responsible for a broad array of diverse cephalic, pharyngeal and cardiac connective tissues and organs in amniotes [54]. The predominant contributions of CNCC to pericytes and smooth muscle of the aortic sac, pharyngeal arteries, aorticopulmonary septum and OFT valves, as well as to the anterior cardinal veins and venous pole, were determined by these means [51,55] (Figure 2B,C). Cardiac parasympathetic ganglia are likewise derived from vagal CNCC [56,57]. Quail-chick chimeras allowed the more recent demonstration that vSMC of the coronary arteries and of the smaller vessels of the upper interventricular septum are derived from pre-otic hindbrain neural crest, although some of these also originate from the proepicardium [58].

Additional appreciation for these migratory pathways and the molecularly controlled extent of cellular admixture came from the contributions of tools in mouse genetics to lineage-trace NCC subsets. *Wnt1-Cre*-driven conditional recombination of a floxed cassette that lifted the transgenic brake for transcription of beta-galactosidase, or later of fluorescent protein transgenes, validated the participation of CNCC derivatives to the composition of the cardiac OFT, tricuspid valves, the vascular smooth muscle of proximal coronary arteries and their septal branches and many surrounding tissues [38,39,58,59,60,61] (Figure 2A, D–F). Tracing lineage analysis using *Krox20-Cre* mice has revealed that a subset of CNCC migrated from rhombomere 5–6 region contributes to semilunar valve development [40]. Furthermore, it became possible to examine how the cells were organized in genetic backgrounds that induce OFT malformations, such as the “rescued” *Raldh2*^−/−^ mutant, where judiciously administered maternal RA can prolong fetal life into mid-gestation as a model of PTA or vitamin A deficiency syndrome [62]. In this example, CNCC are present and viable, but more haphazardly oriented and in an inappropriate location within mutant OFT cushions; subsequently, endocardial contact alone is not enough to ensure fusion and septation but requires close proximity of functionally organized CNCC derivatives (Figure 2B).

Like the pSHF, it is less recognized that a subpopulation of CNCC simultaneously invests the venous pole of the heart when shaping the OFT. This was demonstrated by Poelmann and Gittenberger-de Groot [55] in chicken embryos using a replication-incompetent retrovirus expressing *LacZ* to infect pre-migratory CNCC and trace their cellular progeny to the dorsal mesocardium, the inter-atrial septum, and the presumptive cardiac conduction system and semilunar valves. Lineage tracing in mice confirmed that multipotent CNCC also contribute directly to some proximal conduction system elements of the posterior inter-nodal tract, His bundle and bundle branches [61]. Subsequent studies by independent authors using lineage tracing or single-cell transcriptomics have validated the differentiation of CNCC into some cells of the conduction system or even proximal IVS and OFT myocardial cells [63,64,65].

That CNCC can differentiate into some cardiomyocytes in the mammalian heart is less surprising in light of its substantial contributions to this lineage in the zebrafish OFT but also in its atrium, AV junction and ventricle [66,67]. Only pre-somitic neural crest differentiates into myocardium in this animal that lacks a divided systemic and pulmonary circulation [67]. By contrast, in the amphibian *Xenopus laevis*, the SHF provides OFT cells and presumably also its incomplete septum; CNCC, as defined by the experimental techniques used, seemed to not be required for septal cushion formation [68]. However, after CNCC ablation or labeling, the residual Xenopus OFT septum expresses the Sox8 transcription factor in common with facial neural crest-derived mesenchyme, and recently, Sox8 has been shown in the chick to be a specific core member of the gene regulatory network (GRN) of CNCC [69]. It therefore is possible that the authors examining this question in the amphibian model missed a distinct CNCC contribution to its proximal heart, or that the GRN was co-opted by mesenchyme of SHF origin to accomplish an analogous purpose. CNCC are present in all vertebrate models that have been examined to date, but their precise roles and contributions are organism-specific, reflecting their remarkable plasticity. An interesting example is the crocodile, one of the few reptiles examined with a nearly complete separation of the systemic from the pulmonary circulation. A large septal cushion provides valvular leaflets to each of the three arterial trunks—the right aorta is systemic and has a bicuspid valve, while the left aorta, which irrigates the viscera, and the pulmonary artery have tricuspid valves. Leaflets also serve to block a foramen between the two aortae during systole, reducing pulmonary pressure and favoring blood flow to the brain, even under prolonged diving conditions, Remarkably, the interventricular septum between the left and right OFT remains incomplete until at least birth and shows chondrification within the condensed mesenchyme closest to the OFT, considered by the authors to be most likely of CNCC origin [70]. Enabling new capacities for tissue differentiation and patterning has conferred an obvious evolutionary advantage for adaptation of the head, heart and lungs to a wide range of aqueous or terrestrial environments, from benthic pressures to high-altitude hypoxia. It is important to keep such species-specific adaptations in mind when extrapolating the findings from experimentally tractable models like chick and mouse to human cardiac development.

Orofacial, ear and neck and cardiac OFT anomalies are all heavily reliant on cephalic and vagal neural crest cell influx and remodeling and can be phenocopied in animal models [46], and they are often associated with birth defects in humans. Characteristic signs of genetic conditions such as chromosome 21 trisomy or 22q11.2 microdeletion, they are also frequently induced by the environment during pregnancy (for example, fetal alcohol spectrum disorders and vitamin A or folic acid deficiencies). Both thymic and cardiac OFT malformations such as PTA in the 22q11 microdeletion syndromic spectrum can be reproduced by the ablation of the posterior hindbrain neural folds that would have given rise to CNCC [71], the first major demonstration that paracrine signaling to or from this mesenchymal lineage is critical for the development of organs not themselves primarily constructed from NCC (reviewed in Reference [46]). Severe craniofacial and cardiac malformations are also associated in mice with ectopic expression of *Hoxb1* in *Wnt1-Cre*-recombined CNCC, leading to combinations of hypoplastic posterior pharyngeal arches, interrupted aortic arch (IAA) type B, DORV or OA in addition to cleft palate. CNCC rostrocaudal identity, as determined in part by *Hox* gene expression combinations, impacts its competence to pattern the distal branches of the aortic arches and to influence the rotation of the OFT during its septation [72,73].

As CNCC migrate through the ventral mesenchyme of the posterior pharyngeal arches and the mesoderm of the SHF in order to partition, shape and align the great arteries of the OFT, they interact with all three germ layers through both direct cell contact and through paracrine signaling. One such factor needed to maintain CNCC in this area (and elsewhere in the head) is Fgf8, of both ectodermal and endodermal origin. Like *Tbx1* and *Raldh2* mutants, hypomorphic murine *Fgf8* mutants also phenocopy multiple symptoms of human 22q11.2 microdeletion, including PTA, IAA type B, VSD and patent ductus arteriosus (see below). Insufficient Fgf8 leads to massive circumpharyngeal CNCC apoptosis, as shown by co-labeling neural crest by TUNEL to detect apoptotic cells and anti-AP-2α as they migrate from the post-otic hindbrain into the lateral developing fourth and sixth arches [74]. 

Failure of normal rotation of OFT myocardium may underlie major forms of CHDs. Rotation of the myocardial wall of the OFT is intimately linked to the influx of CNCC. Mammalian embryos undergo a counterclockwise rotation of the OFT [4,75]. The continued addition of right-sided myocardium during normal development results in movement without spiralization of the OFT, in which the pulmonary trunk orifice is pushed in a rightward and anterior direction, a mechanism referred to as ‘‘pulmonary push’’ [76]. In *Lrp2* knockout embryos, the bulk of the SHF population remains in a mid-sagittal position after insufficient pulmonary push [77]. *Lrp2* encodes megalin, a multiligand endocytic co-receptor that is necessary to transduce retinol, Shh and Fgf8, among other environmental signals. Its full knockout leads to PTA in mice, and occasionally in humans, by exhausting the pool of OFT myocardial progenitors through premature differentiation [78]. However, compound heterozygous *LRP2* missense variants have also been associated with hypoplastic left heart syndrome in humans [79], implying that other factors also mediate lateralized SHF contributions to the myocardial base of the truncal arteries.

## 3. Retinoic Acid Signaling

### 3.1. RA Is Required for the Differentiation of Heart Progenitors into the Myocardium of the OFT

Several conotruncal heart defects have been reported in fetal vitamin A deficiency syndrome. Either an excess or lack of retinoic acid (RA), the most active derivative of Vitamin A, causes conotruncal heart defects, indicating that properly controlled RA dosage and signaling is required for normal OFT development [80,81]. This is corroborated by the frequent OFT and aortic arch malformations associated with the complex Matthew-Wood syndrome (also termed Microphthalmia, syndrome 9, Online Catalog of Human Genes and Genetic Disorders (OMIM) 601186), due to inactivating mutations of the *STRA6* gene, which encodes a transmembrane receptor allowing vitamin A cellular uptake [82,83,84,85]. Many heart phenotypes that occur in either fetal vitamin A deficiency syndrome or in excessive maternal exposure to vitamin A or its analog, isotretinoin, are recapitulated in animal models that are mutant for RA nuclear receptors [86]. Conotruncal defects are observed in retinoid acid receptors RARα/RARβ and RARα/RARγ double-mutant hearts and also in hearts with double null alleles in one RXR (α, β, or γ) and one RAR [87,88,89,90,91]. In RA receptor mutant mice (RARα1/RARβ double mutant), the OFT is shortened and misaligned [91]. PTA occurs with high incidence in RARα1/RARβ double mutant mice. In these mutants, truncated OFT and abnormal conotruncal cushion ridges are observed. In RXRα-deficient hearts, OFT septation defects are observed, but there are no apparent defects in RXRβ or RXRγ single mutants [87,88,89,90,92,93]. Therefore, RXRα is the major RXR regulating OFT development.

RA mediates SHF patterning in part through the generation and maintenance of heart progenitors in the aSHF [23,24]. The SHF deficiency in RA receptor-null embryos results in a shortened OFT and thereby in alignment defects. As a related consequence, the tissue of the shortened OFT is mis-specified when septation is initiated [91]. Raldh2, a critical enzyme for the synthesis of endogenous RA in the early embryo, is expressed in caudal pharyngeal mesoderm. Analysis of aSHF marker gene expression, including *Fgf8* and *Tbx1*, in *Raldh2*-mutant embryos shows that RA signaling defines both the posterior boundary of the aSHF [94,95] and its anteromedial boundary with the FHF in the zebrafish [96]. Consistent with these observations, an excess of RA, cardioteratogenic in humans, results in downregulation of *Tbx1* expression in avian embryos [97]. Expression of *Raldh2* and one or more of the *Cyp26* genes, encoding a RA degradation enzyme, is often complementary within a tissue. This establishes a RA gradient between adjacent high and low RA-expressing regions [98,99,100].

### 3.2. Cell-Autonomous RA Signaling Is Dispensable for the Migration and Fate Differentiation of CNCC

Experimental ablation of sources of CNCC induces many of the cardiac and extracardiac malformations found in vitamin A deficiency [69,96]. However, neural crest-targeted deletion of Rxrα/Rarα1 leads to normal cardiovascular morphology, indicating that cell-autonomous RA signaling through this specific combination is dispensable for CNCC migration and differentiation [101]. Excess RA signaling, on the other hand, blocks both CNCC migration and proliferation by preventing c-Jun N-terminal kinase (JNK) phosphorylation in response to such growth factors as Platelet Derived Growth Factor-AA (PDGF-AA) and antagonizing subsequent activation of the transcriptional regulator c-Jun in the AP-1 transcriptional complex [102]. Knockout mice for c-jun invariably develop PTA and abnormal aortic arch remodeling [103]. This AP-1 component is also necessary in all other *Isl1*-expressing OFT cell lineages to prevent less penetrant aortic or pulmonary valve defects, VSD or DORV [104].

### 3.3. RA Signaling Defects Are Associated with DiGeorge Syndrome

*TBX1* is within the most candidate gene-relevant interval in human 22q11.2 deletion syndrome (DiGeorge/velocardiofacial syndrome), and *Tbx1*-mutant mice show similar phenotypes to those of human 22q11.2, in particular TOF [105]. Studies have shown the importance of the Tbx1-Fgf8-Isl1 network in the regulation of OFT development [106]. Conditional deletion of *Fgf8*-in the Nkx2.5-Cre lineage results in truncated OFT, suggesting that Fgf8 plays a role in the proliferation and differentiation of heart progenitors [106]. Mutant mice with a hypomorphic allele of *Raldh2* die perinatally because of cardiovascular defects, similar to those observed in human 22q11.2 deletion syndrome. In this RA-deficient model, anterior pharyngeal arches develop normally, whereas the absence of hypoplastic posterior pharyngeal arches leads to impaired development of the distal OFT and thereby to TOF, DORV and PTA. CNCC migrate toward the posterior pharyngeal arches but fail to invest them [95,107]. In the *Raldh2* mutant, the expression of *Tbx1* is not altered, but the expression levels of its targets *Fgf8*, *Isl1*, *Hoxa1* and *Hoxb1* are downregulated in the posterior pharyngeal endoderm/ectoderm, showing RA’s central role in coordinating aSHF development [95,107]. *Raldh2* SHF expression is expanded anteriorly (cranially) in *Tbx1* mutant embryos, whereas the expression of the three *Cyp26s* is downregulated; consequently, RA signaling is increased and expanded in the anterior SHF [108]. In a compound mutant (*Raldh2*^+/−^; *Tbx1*^+/−^), in which RA synthesis is decreased in the Tbx1^+/−^ state, conotruncal heart defects/aortic arch anomalies are partially rescued. Therefore, when RA signaling is upregulated in the SHF of patients with 22q11.2 syndrome or of *Tbx1*-deletion mutant mice, it can exacerbate the phenotype. Likewise, decreased levels of embryonic RA synthesis accelerate recovery from arterial growth delay in a mouse model of DiGeorge syndrome [109]. Differences in perceived levels of embryonic RA may contribute to the variability of great artery anomalies observed in DGS/VCFS patients [109].

## 4. Hox Transcription Factors

Homeobox-containing *Hox* genes are known RA signaling targets that play critical roles in patterning all bilaterian embryos [110]. *Raldh2*-null mice showed that RA regulates not only the expression pattern of *Hox* genes but also thereby the fate differentiation of *Hox*-expressing domains [14]. Anterior *Hox* genes *Hoxa1, Hoxa3* and *Hoxb1* expression define distinct sub-domains within the pSHF that contribute to a large part of the atrial and sub-pulmonary myocardium [14,25]. This indicates that *Hox*-expressing progenitor cells of the pSHF contribute to both poles of the heart tube. Fate mapping and clonal analysis experiments have confirmed that pSHF cells contribute to the OFT, and that sub-pulmonary and inflow tract myocardial cells are clonally related [111,112]. *Hoxa1*, *Hoxa3* and *Hoxb1* are expressed in overlapping sub-populations of cardiac progenitor cells in the pSHF and downregulated prior to differentiation [14]. *Hoxb1-* and *Hoxa1-*expressing progenitor cells located in the pSHF segregate to both cardiac poles, contributing to the inflow tract and the inferior wall of the OFT [14]. Furthermore, a RA-responsive *Hoxa3* enhancer has recently been shown to be expressed in progenitor cells of a subset of OFT myocardium [25]. Interestingly, a *Mesp1*+ subpopulation co-expresses the anterior *Hox* genes *Hoxa1*, *Hoxa2*, *Hoxb1* and *Hoxb2*. This *Hox*-positive subpopulation of cardiovascular progenitors is located at E7.25, close to the primitive streak, suggesting that it corresponds to the last cardiovascular progenitor population to emerge from it [113].

Hoxb1 plays a key role in patterning those cardiac progenitor cells that contribute to both cardiac poles [15]. Progenitor cells expressing *Hoxb1* alone or concomitantly with *Hoxa1* contribute to the proximal dorsal OFT or distal OFT, respectively (Figure 1). In contrast, cardiac progenitors that contribute to the superior wall of the OFT and right ventricle do not transcribe *Hox* genes. Genetic tracing of *Hoxb1+* lineages in *Tbx1-*deficient embryos showed that the deployment of Hoxb1-positive cells during OFT formation is regulated by Tbx1 [114]. A double deletion mutant of *Hoxa1* and *Hoxb1* shows conotruncal heart defects as well as aortic arch anomalies due to both impaired SHF development and CNCC defects [73,115]. A spatially patterned pharyngeal region is necessary for CNCC to complete their migration and differentiation, borne out by many other studies documenting the upstream signals and early impact of pharyngeal mesoderm and endoderm on neural crest fate [116,117].

Spatial mis-expression of *Hoxb1* in the anterior SHF results in hypoplastic right ventricle. Activation of *Hoxb1* in embryonic stem cells arrests cardiac differentiation in vitro, whereas *Hoxb1*-deficient embryos display premature cardiac differentiation [15]. Moreover, ectopic differentiation in the pSHF of embryos lacking both *Hoxb1* and its paralog *Hoxa1* induces atrioventricular septal defects [15]. *Hoxb1* is thus required for normal deployment of SHF cells during OFT development [115].

A RA-responsive enhancer of *Hoxa3* induces reporter (*LacZ*) expression in the murine pharyngeal endoderm as well as a subpopulation of CNCC [25]. Indeed, *Hoxa3*-knockout mice show a constellation of symptoms overlapping that induced by experimental CNCC ablation: missing aortic arteries and thymus, hypoplastic thyroid glands, aortic stenosis, hypoplastic right ventricle, bicuspid pulmonary valve and persistent patent ductus arteriosus (PDA) with atrial dilation [118]. Like SHF progenitors, the rostrocaudal identity and competence of CNCC is largely determined before EMT, as experimental substitution of pre-migratory neural crest expressing additional (trunk) or no (cephalic) *Hox* genes in place of avian CNCC can induce PTA, DORV and VSD, although their competence to differentiate into cardiac ganglionic cells is less origin-restricted [119]. It is possible to confer CNCC and mesectodermal competence on trunk-level neural crest by the ectopic expression of the *Sox8*, *Tgif1* and *Ets1* transcription factors from the newly identified CNCC-specific GRN [69]. Single-cell transcriptomic studies have found that the post-otic CNCC in mouse embryos is uniquely characterized by *Hoxd3*, *Pax3* and *Nkx1-2* expression, while both *Hoxb1*+ and *Hoxd3*+ CNCC subpopulations co-express *Hoxb2*, *Hoxb3* and *Meis1* [64]. TALE-superclass transcription factors (three-amino acid length extension) such as Pbx1/2/3 or Meis1/2, which are co-factors of anterior Hox proteins, are also expressed in cardiac progenitors, implying a wider role for HOX/TALE complexes during SHF development [120,121,122].

## 5. Other Signaling Pathways

The Fgf, Bmp, Shh, Wnt, Pdgf, endothelin, nitric oxide, planar cell polarity and other signaling cascades have been implicated in the direct or indirect maintenance of CNCC and their subsequent influence on the other cell lineages of the OFT, but a complete discussion of their roles is beyond the scope of this review. However, a general theme appears to be that the molecular signals we identify as important in the specification and function of any given lineage in the cardiac OFT usually have a direct role to play in derivatives of the other lineages as well.

## 6. Future Direction and Clinical Implications

How environmental influences impact the molecular mediators of cardiac OFT development has immediate clinical repercussions. Diabetes mellitus during pregnancy, a frequent condition, increases the risk for congenital heart disease in the offspring, primarily affecting OFT and pharyngeal arch arteries. Cardiac anomalies are often observed in fetuses of diabetic animal models, including rightward displacement of the aorta, DORV and PTA combined with VSD due to a misaligned outlet septum [123]. These defects are strikingly like the DiGeorge syndrome in humans, which has indeed occurred in children of diabetic mothers together with overrepresentation of PTA and DORV. Multiple malformations associated with defective CNCC development in the offspring of diabetic animal models show morphological similarities to those seen in humans [123]. In chicken embryos, exposure of CNCC to elevated D-glucose before their emigration reproducibly induces congenital heart malformations in the OFT and pharyngeal arch arteries, mediated in part by oxidative stress [124]. Mammalian maternal diabetes results in transient and localized alterations in glycation products, VEGF expression and Smad2 phosphorylation, overlapping with those regions of the developing heart that are most sensitive to diabetes-induced CHD [125]. Interaction between genetic and non-genetic factors in epigenetic modifications including DNA methylation and histone modification are likely involved. 

Single-cell RNA sequencing has been applied to study the cellular diversity of heart progenitors [126,127] or embryonic or adult hearts at the whole-organ level [128,129,130,131], allowing efficient examination of developmental lineage trajectories [44]. For example, this type of analysis has identified the transcription factor *Hand2* as a specifier of OFT cells, where examining *Hand2*-null embryos at multiple time points demonstrated failure of OFT myocardial cell specification. Loss of *Hand2* in mouse embryos also leads to dysregulation of RA signaling and disruption of cardiac progenitor patterning [126]. To date, there are few examples of such population analyses of cell states and regulatory networks in diseases with OFT malformations. The near future will certainly supply multi-omics studies to complement the experimental embryology work of the last few decades, to not only know the many lineages and molecular influences needed to shape the vulnerable conotruncal region of the human heart but to enable promising perspectives in prevention, early diagnosis and increasingly effective surgical reconstructions.

## Data Availability

Not applicable.
